# Differences in ambulatory care fragmentation by race

**DOI:** 10.1186/s12913-021-06133-9

**Published:** 2021-02-17

**Authors:** Lisa M. Kern, Mangala Rajan, Lisandro D. Colantonio, Evgeniya Reshetnyak, Joanna Bryan Ringel, Paul M. Muntner, Lawrence P. Casalino, Laura C. Pinheiro, Monika M. Safford

**Affiliations:** 1 Weill Cornell Medicine, 420 East 70th Street, Box 331, New York, NY 10021 USA; 2 University of Alabama at Birmingham, Birmingham, AL USA

**Keywords:** Ambulatory care, Medicare, Race factors

## Abstract

**Background:**

More fragmented ambulatory care (i.e., care spread across many providers without a dominant provider) has been associated with more subsequent healthcare utilization (such as more tests, procedures, emergency department visits, and hospitalizations) than less fragmented ambulatory care. It is not known if race and socioeconomic status are associated with fragmented ambulatory care.

**Methods:**

We conducted a longitudinal analysis of data from the REasons for Geographic and Racial Differences in Stroke (REGARDS) study, using the REGARDS baseline visit plus the first year of follow-up. We included participants ≥65 years old, who had linked fee-for-service Medicare claims, and ≥ 4 ambulatory visits in the first year of follow-up. We used Tobit regression to determine the associations between race, annual household income, and educational attainment at baseline and fragmentation score in the subsequent year (as measured with the reversed Bice-Boxerman Index). Covariates included other demographic characteristics, medical conditions, medication use, health behaviors, and psychosocial variables. Additional analyses categorized visits by the type of provider (primary care vs. specialist).

**Results:**

The study participants (*N* = 6799) had an average age of 73.0 years, 53% were female, and 30% were black. Nearly half had low annual household income (<$35,000) and 41% had a high school education or less. Overall, participants had a median of 10 ambulatory visits to 4 providers in the 12 months following their baseline study visit. Participants in the highest quintile of fragmentation scores had a median of 11 visits to 7 providers. Black race was associated with an absolute adjusted 3% lower fragmentation score compared to white race (95% confidence interval (2% lower to 4% lower; *p* < 0.001). This difference was explained by blacks seeing fewer specialists than whites. Income and education were not independent predictors of fragmentation scores.

**Conclusions:**

Among Medicare beneficiaries, blacks had less fragmented ambulatory care than whites, due to lower utilization of specialty care. Future research is needed to determine the effect of fragmented care on health outcomes for blacks and whites.

**Supplementary Information:**

The online version contains supplementary material available at 10.1186/s12913-021-06133-9.

## Background

“Fragmented” ambulatory care occurs when patients see many ambulatory providers with no single provider accounting for a substantial portion of visits [1]. Although seeing many providers may be clinically appropriate, it creates challenges, because providers do not consistently communicate with each other regarding their common patients [2]. As a result, fragmented ambulatory care increases the risk that clinically important information will be missing at the point of care [3]. More fragmented care has been associated with lower rates of receipt of recommended care [4, 5], more drug-drug interactions [3, 6], more radiology tests [7], more procedures [8], more emergency department visits [1, 9–11], and more hospitalizations [1, 9, 12], compared to less fragmented care. Whether the frequency of highly fragmented care varies with race, income, and educational attainment is not known.

Previous work has shown that blacks, individuals with low income, and individuals with low educational attainment are at high risk for receiving lower rates of receipt of recommended care, compared to whites, those with high income, and those with high educational attainment [13, 14]. Thus, one might hypothesize that blacks, those with low income, and those with low educational attainment are likely to have more fragmented care than whites, those with high income, and those with high educational attainment.

However, blacks, those with low income, and those with low educational attainment have also been shown to have less access to specialist care than whites, those with high income, and those with high educational attainment [15, 16]. This disparity has been found even when there are no differences in health insurance [15]. Therefore, blacks, those with low income, and those with low educational attainment might have less fragmented care than their counterparts.

We sought to determine the associations of race, income, and educational attainment with ambulatory care fragmentation, adjusting for potential confounders. We also sought to determine whether any differences in fragmentation were driven by differences in specialty utilization.

## Methods

### Overview

We conducted a secondary analysis of data from the nationwide REasons for Geographic and Racial Differences in Stroke (REGARDS) study [17]. We used participant characteristics determined during the REGARDS study baseline visit and assessed ambulatory utilization in the 12 months following the baseline visit. Institutional Review Boards (IRBs) of the participating institutions approved the protocol. All participants provided written informed consent.

### Setting, population, and data sources

Between 2003 and 2007, 30,239 community-dwelling black and white adults ≥45 years old were enrolled in the REGARDS study [17]. As previously described, the REGARDS sample was selected from commercially available nationwide lists of individuals, divided into strata by geography, race, and sex [17]. The study involved oversampling of participants living in the Southeastern U.S., balanced sampling of black and white individuals, and balanced sampling of men and women [17]. Potential participants were contacted first by mail and then by phone [17]. Exclusion criteria included race other than black or white, Hispanic ethnicity, active treatment for cancer, medical conditions that would preclude long-term participation, cognitive impairment judged by the trained telephone interviewer, residence in or inclusion on a waiting list for a nursing home, and inability to communicate in English [17].

The organizations involved in data collection for the REGARDS study include: an Operations Center and Survey Research Unit (SRU) at the University of Alabama at Birmingham, a central laboratory at the University of Vermont, an electrocardiogram reading center at Wake Forest University, a company that conducted at-home visits (Examination Management Services Inc. [ESMI]), and a medical monitoring and stroke adjudication center at Alabama Neurological Institute, Inc. [17] An Executive Committee consisting of the principal investigator (PI) at each study center, plus a representative from the National Institute of Neurological Disorders and Stroke, assists the REGARDS PI at the University of Alabama at Birmingham with the scientific direction of the REGARDS study [17]. This Executive Committee reviewed all study methods and data collection protocols, which were approved by the participating institutions’ IRBs [17].

Baseline data collection involved computer-assisted telephone interviews and in-home visits with a physical examination, blood test, urine test, electrocardiogram, and medication inventory [17]. This data collection was conducted by approximately 100 trained telephone interviewers and approximately 6500 trained ESMI examiners [17]. Performance by interviewers and examiners was monitored by SRU and ESMI, respectively [17]. Details regarding the data collection instruments, which included many previously validated measures, have been published [17].

For our primary analysis, we used data from the REGARDS baseline in-home study visit. As a sensitivity analysis, we used data from the REGARDS second in-home study visit, which took place between 2013 and 2016, approximately 10 years after the baseline visit, as well as data on adjudicated cardiovascular events that had occurred by the time of the second in-home study visit [18, 19]. For all analyses, we also used participants’ linked Medicare claims for the 12 months after each REGARDS in-home study visit [20].

### Variables

Participant characteristics were collected by the REGARDS study and included: demographics (age, sex, race, marital status, annual household income, educational attainment, geographic region, and rural/urban setting), medical conditions (hypertension, dyslipidemia, diabetes, atrial fibrillation, myocardial infarction, and stroke), medications (total number of medications taken in the past 2 weeks; and use of antihypertensive medication, insulin, and/or statin), health behaviors (cigarette smoking status, alcohol use, and exercise frequency), psychosocial variables (being the primary caretaker for another individual, having seen any close friends or relatives in the past month, and depressive symptoms), physiologic variables (body mass index; systolic blood pressure; total, low density lipoprotein and high-density lipoprotein cholesterol; serum glucose; estimated glomerular filtration rate; urinary albumin-to-creatinine ratio; and high sensitivity c-reactive protein), and self-rated health. The definitions of these variables, which incorporate previously validated definitions [21–26], can be found in Appendix 1.

We used Medicare claims to identify ambulatory visits for the 12-month period following the REGARDS study visit. Ambulatory visits were defined using a modified National Committee for Quality Assurance (NQCA) definition [27] that was restricted to Clinical Procedure Terminology (CPT) codes for in-person, evaluation-and-management visits for adults in an office setting [7]. The NCQA definition of ambulatory visits does not include emergency department visits. We identified ambulatory providers by considering the Unique Provider Identification Numbers (UPINs) in the claims for the ambulatory visits. For each participant, we determined the percentage of visits with the most frequently seen provider.

For each participant, we calculated a fragmentation score using the Bice-Boxerman Index (BBI) [28], which has been previously validated [9, 12, 29]. This index captures both “dispersion” (the spread of ambulatory visits across providers) and “density” (the relative share of visits by each provider) [30]. Patterns of care characterized by high dispersion (many providers) and low density (a relatively low proportion of visits by each provider) receive worse scores (indicating more fragmentation) than patterns with low dispersion and high density. The original BBI ranges from 0 (each visit with a different provider) to 1 (all visits with same provider). To facilitate interpretation, we reversed the index, calculating 1 minus BBI, so that higher scores reflected more fragmentation [1, 7, 11]. Appendix 2 shows the formula for the BBI. Appendix 3 provides examples of ambulatory care patterns and their corresponding BBI scores.

For additional analyses, we categorized providers as primary care or specialty care, using the National Plan and Provider Enumeration System (NPPES) [31].

### Statistical analysis

We included participants ≥65 years old whose REGARDS study data were linked to Medicare claims at baseline. We excluded participants who did not have fee-for-service Medicare, did not have continuous coverage for 12 months following baseline, or died within one year after the baseline visit. We excluded participants who qualified for Medicare on the basis of end-stage renal disease, as utilization patterns for these beneficiaries are substantively different from those of other beneficiaries [32]. We also excluded those who had ≤3 ambulatory visits in the first year of observation, as calculating fragmentation scores with fewer than 4 visits can yield unstable estimates [12]. Finally, we excluded (and then later re-included in a sensitivity analysis) participants who had fragmentation scores that were equal to the ends of the scale (equal to 0.00 or 1.00), as these scores are relatively uncommon and represent ambulatory care patterns that may violate underlying trends [7]. For example, a beneficiary can have a score equal to 1.00 if he or she has 4 visits with 4 different providers, but this is not conceptually “more fragmented” than a beneficiary who has 9 visits with 6 different providers and a fragmentation score of 0.92 [7].

We used descriptive statistics to characterize the final study sample. We compared those included in the study to those who had been excluded on the basis of having ≤3 ambulatory visits, using t-tests, chi-squared tests, and Wilcoxon rank sum tests.

We divided the study sample into quintiles based on their fragmentation scores. We determined the median number of visits, providers, proportions of visits with the most frequently seen provider, and fragmentation scores within each quintile. We calculated *p*-values for trend across quintiles for each of these measures of ambulatory utilization.

To explore the unadjusted associations of race, income, and education with fragmentation scores, we calculated the percentage of individuals with a given characteristic (black race; annual household income <$35,000; and high school education or less) within each fragmentation quintile. We then calculated *p*-values for trend across quintiles. To facilitate interpretation, we generated descriptive statistics of ambulatory utilization (visits, providers, percentage of visits with the most frequently seen provider, and fragmentation score) stratified by race, income, and education. We further stratified visits and providers by primary care vs. specialty care. We compared ambulatory utilization patterns across subgroups by race, income, and education, using non-parametric Wilcoxon two-sample tests.

We used Tobit models to determine whether race, income, and education were associated with fragmentation scores. We used Tobit models instead of linear models, because the possible values for fragmentation were bounded. Interpretation of Tobit models is the same as for linear models; coefficients represent the absolute amount of change in the fragmentation score. Because the fragmentation score is on a scale from 0.01 to 0.99, changes in the absolute fragmentation score can be multiplied by 100 to yield an equivalent percent change in the fragmentation score. Unadjusted models considered race, income, and education separately. Model 1 adjusted for race, income and education in the same model. Model 2 added adjustment for age, sex, marital status, geographic region, and rural geography. Model 3 included all variables in Model 2 plus medical conditions and medications. Model 4 included all variables in Model 3 plus health behaviors, psychosocial variables, physiologic variables, and self-rated health.

Analyses were conducted with SAS (version 9.4, Cary, NC). *P*-values < 0.05 were considered statistically significant.

## Results

### Study sample

Of the 30,239 participants in the REGARDS study at baseline, 14,961 (49%) were ≥ 65 years old (Appendix 4). Of those, 8865 (59%) had Medicare fee-for-service, continuous coverage for 12 months, were alive one year after baseline, and did not have end-stage renal disease. Of those, 7120 (80%) had ≥4 ambulatory visits. There were 321 participants (4.5%) who were excluded (and later re-included) for having fragmentation scores equal to 0.00 or 1.00. Thus, the final sample size for the main analysis was 6799.

Participants excluded for having ≤3 ambulatory visits were younger, less likely to be female, and more likely to be married than those included in the final sample. Those excluded for having ≤3 ambulatory visits were not statistically significantly different from those included in terms of race, income, and education. Other differences are shown in Table 1.

**Table 1:** Participant characteristics at baseline and ambulatory care utilization in the subsequent 12 months, comparing those included (with ≥4 visits) to those excluded (with ≤3 visits)

Characteristic	Included (≥4 visits) *N* = 6799	Excluded (≤3 visits) *N* = 1745	*P*-value
Demographic characteristics			
Age, years, mean (SD)	73.0 (5.9)	72.0 (5.5)	< 0.0001
Sex, female, N (%)	3573 (53)	734 (42)	< 0.0001
Race, black, N (%)	2042 (30)	561 (32)	0.09
Marital status, married, N (%)	3962 (58)	1064 (61)	0.04
Annual household income, <$35,000, N (%)	3251 (48)	862 (49)	0.16
Education, high school or less, N (%)	2779 (41)	691 (40)	0.40
Geographic region, N (%)			
Stroke Belt^a^	2428 (36)	641 (37)	0.001
Stroke Buckle^b^	1628 (24)	347 (20)	
Neither Stroke Belt nor Stroke Buckle	2743 (40)	757 (43)	
Rural geography, N (%)	612 (10)	179 (11)	0.08
Medical conditions^c^			
Hypertension, N (%)	4543 (67)	976 (56)	< 0.0001
Dyslipidemia, N (%)	4246 (65)	994 (59)	< 0.0001
Diabetes, N (%)	1580 (24)	264 (16)	< 0.0001
Atrial fibrillation, N (%)	816 (12)	92 (5)	< 0.0001
Myocardial infarction, N (%)	1168 (18)	234 (14)	< 0.0001
Stroke, N (%)	576 (9)	106 (6)	< 0.001
Medications			
Number of medications, median (25th, 75th percentiles)	7 (4, 10)	4 (2, 7)	< 0.0001
Anti-hypertensive medication, N (%)	4054 (61)	771 (44)	< 0.0001
Insulin use, N (%)	412 (6)	50 (3)	0.04
Statin use, N (%)	2841 (40)	527 (30)	< 0.001
Health behaviors			
Current smoker, N (%)	566 (8)	211 (12)	< 0.0001
Alcohol use, moderate or heavy, N (%)	2242 (34)	617 (36)	0.02
Exercise frequency, 0 times per week, N (%)	2467 (37)	564 (33)	< 0.001
Psychosocial variables			
Cares for a family member with a chronic illness or disability, N (%)	715 (11)	177 (10)	0.65
Lack of social support, N (%)	274 (4)	81 (5)	0.24
Depressive symptoms, N (%)	620 (9)	118 (7)	< 0.01
Physiological variables			
Body mass index, kg/m^2^, median (25th, 75th percentiles)	27.6 (24.6, 31.3)	27.3 (24.4, 30.5)	< 0.01
Systolic blood pressure, mm Hg, median (25th, 75th percentiles)	129 (119, 139)	129 (120, 140)	0.26
Total cholesterol, mg/dL, median (25th, 75th percentiles)	183 (159, 210)	190 (166, 220)	< 0.0001
Low-density lipoprotein cholesterol, mg/dL, median (25th, 75th percentiles)	105 (85, 129)	114 (92, 137)	< 0.0001
High-density lipoprotein cholesterol, mg/dL, median (25th, 75th percentiles)	49 (40, 61)	49 (39, 60)	0.68
Glucose, mg/dL, median (25th, 75th percentiles)	95 (87, 109)	94 (88, 105)	0.04
Estimated glomerular filtration rate, mL/min/1.73 m^2^, median (25th, 75th percentiles)	78.1 (63.6, 89.5)	83.0 (71.0, 90.7)	< 0.0001
Urinary albumin-to-creatinine ratio, mg/g, median (25th, 75th percentiles)	9.1 (5.3, 20.2)	7.4 (4.8, 15.4)	< 0.0001
C-reactive protein, mg/L, median (25th, 75th percentiles)	2.1 (0.9, 4.8)	1.8 (0.9, 4.2)	< 0.001
Self-rated health			
Self-rated general health, N (%)			
Excellent	966 (14)	421 (24)	< 0.0001
Very good	2079 (31)	609 (35)	
Good	2495 (37)	511 (29)	
Fair	1022 (15)	165 (10)	
Poor	224 (3)	36 (2)	
Ambulatory utilization			
Number of ambulatory visits, median (25th, 75th percentile)	10 (7, 15)	2 (0, 3)	< 0.0001
Number of ambulatory providers, median (25th, 75th percentile)	4 (3, 6)	1 (0, 2)	< 0.0001
Proportion of visits with the most frequently seen provider, median (25th, 75th percentile) ^d^	0.44 (0.33, 0.58)	–	–
Fragmentation score (reversed Bice-Boxerman Index), median (25th, 75th percentile) ^d^	0.78 (0.65, 0.86)	–	–

^a^ Stroke Belt = North Carolina, South Carolina, Georgia, Tennessee, Mississippi, Alabama, Louisiana, and Arkansas (except for 153 coastal counties that constitute the Stroke Buckle) [17]

^b^ Stroke Buckle = 153 coastal counties in North Carolina, South Carolina, and Georgia [17, 18]

^c^ See Appendix 1 for detailed definitions of variables

^d^ Proportion of visits with the most frequently seen provider and fragmentation scores are not reliable if based on ≤3 visits [12]

The participants in the study sample were 73.0 years old on average, half (53%) were female, and 30% were black. More than half (58%) were married. Nearly half (48%) had low annual household income (i.e., <$35,000), and 41% had a high school education or less. Additional participant characteristics are shown in Table 1.

### Ambulatory utilization

Overall, participants had a median of 10 ambulatory visits across 4 providers in the 12 months following their baseline visit, the most frequently seen provider accounted for a median of 44% of visits, and the median fragmentation score was 0.78. Participants in the quintile with the most fragmented care had a median of 11 ambulatory visits to 7 providers, with the most frequently seen provider accounting for 28% of visits and a median fragmentation score of 0.90. (Table 2).

**Table 2:** Ambulatory utilization over a 12-month period stratified by fragmentation quintile^a^ (*N* = 6799)

Fragmentation quintile	N	Fragmentation score (median)	Number of visits (median)	Number of providers (median)	Proportion of visits with the most frequently seen provider (median)
1 (least fragmentation)	1419	0.50	8	2	0.73
2	1222	0.68	10	4	0.56
3	1380	0.78	10	4	0.44
4	1369	0.83	11	5	0.26
5 (most fragmentation)	1409	0.90	11	7	0.28
P for trend		< 0.0001	< 0.0001	< 0.0001	< 0.0001

^a^Healthcare fragmentation quintiles were derived from reversed Bice-Boxerman scores (0.01 to 0.99)

**Fig. 1:**
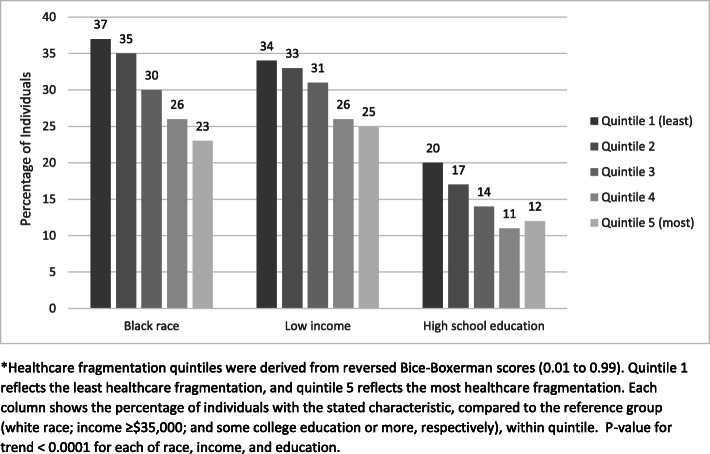
Percentage of individuals within each quintile of fragmentation scores by race, income, and education (*N* = 6799)*

Black participants were less likely to have highly fragmented care than white participants (*p* < 0.001 for trend) (Figure 1). Those with annual incomes <$35,000 were less likely to have highly fragmented care than those with annual incomes ≥$35,000 (*p* < 0.0001 for trend). Those with a high school education or less were less likely to have highly fragmented care than those with a college education or more (p < 0.0001 for trend).

These differences in care patterns by race, income, and education were not explained by any differences in the total number of ambulatory visits by the participants. That is, black participants, those with low income, and those with low education all had more visits than white participants, those with higher income, and those with more education (*p* < 0.05 for each comparison, Table 3). These differences were also not explained by primary care utilization, with black participants, those with low income, and those with low educational attainment having more primary care visits and more primary care providers than their counterparts.

**Table 3:** Ambulatory care patterns overall and stratified by race and socioeconomic status^a^

	Number of visits, mean (sd)			Number of providers, mean (sd)			Percentage of visits with the most frequently seen provider (*N* = 6799)	Fragmentation Score,^b^ mean (sd) (*N* = 6799)
	Total (*N* = 6799)	Primary Care (*N* = 6741)	Specialty Care (*N* = 6741)	Total (*N* = 6799)	Primary Care (*N* = 6741)	Specialty Care (*N* = 6741)		
Overall	11.7 (7.1)	5.5 (4.4)	6.2 (5.0)	4.9 (2.4)	1.6 (1.1)	3.2 (2.0)	0.47 (0.17)	0.73 (0.16)
By race								
Black	12.0 (7.0)	6.0 (4.5)	5.8 (4.8)	4.7 (2.4)	1.7 (1.2)	2.9 (1.9)	0.49 (0.17)	0.71 (0.17)
White	11.6 (7.1)	5.3 (4.3)	6.3 (5.1)	5.0 (2.4)	1.6 (1.0)	3.4 (2.0)	0.46 (0.17)	0.75 (0.16)
By income								
< $35,000	12.0 (7.1)	5.8 (4.5)	6.0 (5.0)	4.8 (2.4)	1.7 (1.0)	3.1 (2.0)	0.48 (0.17)	0.72 (0.17)
≥ $35,000	11.3 (6.7)	5.0 (4.1)	6.2 (4.9)	5.0 (2.4)	1.6 (1.0)	3.4 (2.0)	0.46 (0.16)	0.75 (0.15)
By education								
≤ High school	12.0 (7.1)	5.9 (4.5)	6.0 (4.9)	4.8 (2.4)	1.7 (1.1)	3.1 (1.9)	0.48 (0.17)	0.72 (0.17)
≥ Some college	11.5 (7.0)	5.2 (4.2)	6.3 (5.0)	5.0 (2.4)	1.6 (1.0)	3.4 (2.0)	0.46 (0.17)	0.74 (0.16)

^a^The total sample size and the sample sizes for ambulatory care patterns by primary care vs. specialty care are slightly different, because some participants had care patterns with missing provider specialty. All pairwise comparisons within demographic characteristic are statistically significant (*p* < 0.05)

^b^Fragmentation Index is the reversed Bice-Boxerman Index (0.01 to 0.99); higher scores reflect more fragmentation

Differences in care patterns by race, income, and education were explained by differences in the number of visits to specialty providers, with black participants, those with low income, and those with low educational attainment seeing fewer specialists than their counterparts (Table 3). For example, black participants saw an average of 2.9 specialist providers each, while white participants saw an average of 3.4 specialists each (*p* < 0.05).

In an unadjusted model, black race was associated with a 0.039-point lower fragmentation score, compared to white race (*p* < 0.001, Table 4). Race persisted as a significant predictor of fragmentation, even after adjustment for co-variates. In the fully adjusted model, black race was associated with a 0.027-point lower fragmentation score, equivalent to a 3% lower score, compared to white race (95% confidence interval [CI] 2% lower to 4% lower; p < 0.001). Income and education were associated with lower fragmentation scores in unadjusted models but not fully adjusted models.

**Table 4:** Unadjusted and adjusted associations among race, income, education, and healthcare fragmentation

	Coefficients (95% Confidence Intervals)				
	Unadjusted	Model 1	Model 2	Model 3	Model 4
Race (black vs. white)	−0.039*** (−0.047,-0.031)	−0.033*** (−0.043,-0.024)	− 0.037*** (− 0.047,-0.027)	−0.024*** (− 0.034,-0.013)	−0.027*** (− 0.040,-0.015)
Annual household income (<$35,000 vs. ≥$35,000)	− 0.029*** (− 0.037,− 0.021)	-0.021*** (− 0.030,-0.012)	−0.017*** (− 0.027,-0.007)	−0.013* (− 0.024,-0.003)	−0.010 (− 0.021,0.002)
Educational attainment (high school or less vs. some college or more)	− 0.030*** (− 0.034,-0.019)	−0.013*** (− 0.022,-0.004)	−0.012** (− 0.022,-0.003)	−0.009 (− 0.019,0.001)	−0.002 (− 0.013,0.009)

These models used linear regression to determine associations among race, income, and educational attainment (as independent variables) and healthcare fragmentation (as a dependent variable, using the continuous reversed Bice-Boxerman Index). Negative coefficients indicate lower fragmentation scores than the reference group

Unadjusted models were bivariate models, with race, income, and education in separate models

Model 1 includes race, income, and education in the same model

Model 2 includes Model 1 covariates plus age, sex, marital status, geographic region, and type of geography

Model 3 includes Model 2 covariates plus medical conditions and medications (as shown in Table 1)

Model 4 includes Model 3 covariates plus health behaviors, psychosocial variables, physiological variables, and self-rated health (as shown in Table 1)

*p < 0.05. **p ≤ 0.01. ***p ≤ 0.001

In a sensitivity analysis of the fully adjusted model re-including those with fragmentation scores equal to 0.00 or 1.00, black race was associated with a 0.033-point lower fragmentation score, still equivalent to a 3% lower score, compared to white race (95% CI 2% lower to 5% lower, *p* < 0.001, Appendix 5). In this sensitivity analysis, low income was associated with 2% lower score, compared to higher income (95% CI 0.3% lower to 3% lower, *p* = 0.02). Education was not associated with fragmentation in this sensitivity analysis.

In the sensitivity analysis using data from the second in-home visit, black race was associated with a 0.049-point lower fragmentation score, equivalent to a 5% lower score, compared to white race (95% CI 3% lower to 7% lower). In this sensitivity analysis, income was not associated with fragmentation, but having a high school education or less was associated with a 3% lower fragmentation score, compared to have some college education or more (95% CI 1% lower to 5% lower, Appendix 5).

## Discussion

In this nationwide study of 6799 Medicare beneficiaries, black race was associated with less fragmented ambulatory care. Blacks had fragmentation scores that were 3% lower than whites’ scores (fully adjusted *p* < 0.001). This difference was not explained by any differences in the total number of ambulatory visits, total number of primary care visits, or number of primary care providers. Rather, the difference was driven by the fact that blacks saw fewer specialists than whites (2.9 specialists vs. 3.4 specialists, *p* < 0.05). Income and education were not associated with fragmentation scores in fully adjusted models.

It is difficult to compare this study to previous studies, because previous studies have typically measured specialty access as the percentage of patients who had at least 1 visit with a specialist [15, 16, 33]. Some previous studies measured the percentage of patients who had a least 1 visit with a particular type of specialist (e.g. cardiologist) [16, 34]. Thus, this study adds to the literature by quantifying specialist utilization in more detail, including by race.

It is not known whether the 0.027-point difference in fragmentation score that we found is clinically important. We do know from previous studies that a 0.10-point increase has been associated with a higher risk of hospitalization [12]. However, this amount (i.e., 0.10) was used in part for convenience, given that the original scale is from 0.00 to 1.00, and regression models can easily report the difference in an outcome for each 0.10 change in the score. It is possible that a smaller amount could still be clinically important. More research is needed to understand the relationship between fragmentation and outcomes [5, 35], as well as the amount of change in fragmentation scores that is needed to affect a change in outcomes.

We cannot determine from this study the reasons for the racial differences in care patterns that we observed, and we cannot determine whether blacks having less fragmented care is desirable or undesirable. Although the previous literature suggests that highly fragmented care is often undesirable [1, 7–12], some fragmentation may be clinically appropriate, and it may be that blacks are receiving too little care from specialists, compared to their medical need. A previous study found that black and white Medicare beneficiaries are largely treated by different primary care physicians [36]. In that study, primary care physicians seen by black Medicare beneficiaries were less likely to be board certified and more likely to report not being able to provide high quality care than primary care physicians seen by white Medicare beneficiaries [36]. Primary care physicians seen by black Medicare beneficiaries reported more difficulty obtaining access to specialist physicians and other needed resources [36]. There may be additional reasons for differences in visit patterns by race, including patient preferences. We adjusted for an extensive list of clinical covariates and for geography and rural residence, so those are less likely to explain the differences we observed.

While our study found a consistent association between black race and less fragmentation of care, the results on income and education were less consistent. Although race, income, and education are all considered components of socioeconomic status, they are distinct. Racial disparities in American healthcare are a major problem and have been widely documented, independent of income and education [14]. Yet, differences in ambulatory care and specialist utilization by race have been understudied. Few interventions have tried to change ambulatory care patterns directly [37] and none to our knowledge have tried to change ambulatory care patterns specifically for minority populations.

The consequences of fragmented care are not well understood. Fragmented care may lead to gaps in communication across providers caring for the same patient [3]; however, while previous studies have documented racial disparities in patient-provider communication [38], less is known about racial disparities in provider-provider communication. In addition, while fragmentation of ambulatory care has been shown to be associated with more downstream healthcare utilization (such as hospitalizations), the precise impact on morbidity and mortality is not clear [5, 35]. Moreover, whether such associations vary with race is not yet known.

This study has several strengths, including the nationwide, community-based sample with large numbers of blacks and whites. The study includes clinically detailed potential confounders, which were rigorously collected through standardized protocols. The study also includes linked Medicare claims and previously validated claims-based measures of fragmentation.

This study also has limitations. First, this study does not measure communication across providers and does not measure clinical appropriateness. Second, although it measures racial disparities, it only includes black and white participants; thus, we cannot comment on ambulatory care patterns for Hispanics, Asians, or other racial groups. Third, this study only includes Medicare beneficiaries ≥65 years, potentially limiting generalizability to other payer populations or other age groups.

## Conclusions

In conclusion, among Medicare beneficiaries, blacks had less fragmented ambulatory care than whites, due to lower utilization of specialty care. This finding was present after accounting for income, education, other demographic characteristics, medical conditions, medication use, health behaviors, psychosocial variables, physiologic variables, and self-rated health. This work advances understanding of racial differences in ambulatory care patterns. The next steps in this line of inquiry will be to determine whether fragmentation is associated with health outcomes and, if so, whether any such association varies with race.

## Supplementary Information

**Additional file 1.**

## Data Availability

The datasets used for this study are not publicly available, because this study is part of an ancillary study to a parent study, the REasons for Geographic and Racial Differences in Stroke (REGARDS) study. The ancillary study does not have the ability to grant access to the data.

## Abbreviations

BBI: Bice-Boxerman Index
CPT: Clinical Procedure Terminology
ESMI: Examination Management Services Inc
IRB: Institutional Review Board
NCQA: National Committee for Quality Assurance
NPPES: National Plan and Provider Enumeration System
PI: Principal Investigator
REGARDS: REasons for Geographic and Racial Differences in Stroke study
SRU: Survey Research Unit
UPIN: Unique Provider Identification Number
