# Supplementary material for: Differences in ambulatory care fragmentation by race
Source: BMC Health Serv Res. 2021 Feb 17;21:154. doi: 10.1186/s12913-021-06133-9 (PMC7890852; doi:10.1186/s12913-021-06133-9)
Supplement: Supplementary file 1 — Additional file 1. [file 12913_2021_6133_MOESM1_ESM.pdf]

Appendix 1. Definitions of variables used to describe characteristics of participants, based on data collected by the Reasons for Geographic and Racial Differences in Stroke study[17]

Variable	Definition / Source of information*
Demographic characteristics	
Age	Self-report
Sex	Self-report
Race	Self-report
Marital status	Self-report
Annual household income	Self-report
Educational attainment	Self-report
Geographic region	Participant contact information
Rural / urban setting	Rural/Urban Commuting Area codes[21]
Medical conditions	
Hypertension	Systolic blood pressure ≥140 mm Hg or diastolic blood pressure ≥ 90 mm Hg assessed during the in-home visit, or self-reported use of antihypertensive medication
Dyslipidemia	Use of lipid-lowering medication, total cholesterol ≥ 240 mg/dL, low-density lipoprotein cholesterol ≥160 mg/dL, or high-density lipoprotein cholesterol ≤ 40 mg/dL assessed during the in-home visit
Diabetes	Fasting glucose ≥ 126 mg/dL or non-fasting glucose ≥200 mg/dL assessed during the in-home visit, or self-reported use of oral glucose-lowering medication or insulin
Atrial fibrillation	Self-report of this diagnosis by a physician or nurse, or present on the study electrocardiogram
Myocardial infarction	Self-report of this diagnosis by a physician or nurse, or based on evidence from the study electrocardiogram at baseline (plus adjudicated myocardial infarctions for a sensitivity analysis)
Stroke	Self-report of this diagnosis by a physician or nurse (plus adjudicated strokes for a sensitivity analysis)
Medications	
Total number of medications taken in the past 2 weeks	Assessed by medication inventory
Use of anti-hypertensive medication	Self-report
Use of insulin	Self-report
Use of statin	Assessed by medication inventory
Health behaviors	
Cigarette smoking status	Self-report
Alcohol use	Self-report[22]
Exercise frequency	Self-report

Psychosocial variables	
Being the primary caretaker for another individual	Self-report
Having seen any close friends or relatives in the past month	Self-report
Depressive symptoms	Center for Epidemiologic Studies Depression Scale (CES-D) scores ≥4[23]
Physiologic variables	
Body mass index	Assessed from height and weight measured during the in-home visit physical examination
Systolic blood pressure	Assessed during the in-home visit physical examination
Total cholesterol	Measured via blood test
Low-density lipoprotein cholesterol	Measured via blood test
High-density lipoprotein cholesterol	Measured via blood test
Serum glucose	Measured via blood test
Estimated glomerular filtration rate (eGFR)	Measured via blood test[24]
Albumin-to-creatinine ratio (ACR)	Measured via urine test[25]
High sensitivity c-reactive protein	Measured via blood test
Self-rated health	
Self-rated general health	Self-rated general health, using the first question (SF-1) from the short-form 12 (SF-12)[26]

*See Table 1 for categorical response options for relevant self-reported items. See the Methods section for information on the timing of data collection.

Appendix 2. Formula for the Bice-Boxerman Index (BBI)*[28]

$$BBI = \frac{\left(\sum_{i=1}^{p} n_i^2\right) - n}{n\,(n-1)}$$

where n = total number of visits in the 12-month period
n_i = number of visits to provider i
p = total number of providers

* The BBI is a continuous index that ranges from 0 to 1. We have reversed the direction of the Index, calculating 1 minus BBI, so that higher scores reflect more fragmentation.

Appendix 3. Hypothetical patients, their ambulatory care patterns, and their fragmentation scores*

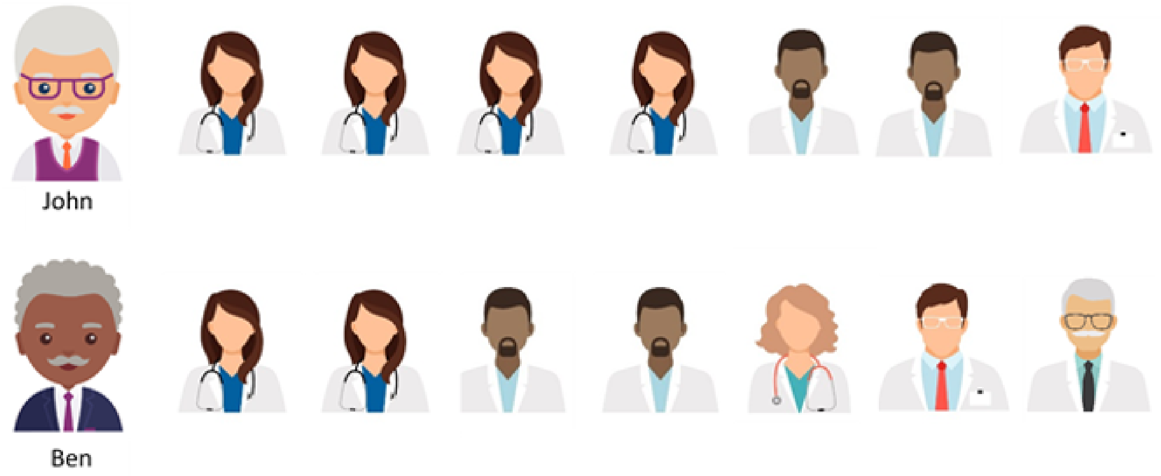

Patient	Number of visits	Number of providers	Bice-Boxerman Index (BBI)	Reversed BBI (rBBI = 1 - BBI)
John	7	3	0.33	0.67
Ben	7	5	0.10	0.90

*Each provider icon represents one visit. If the same provider icon repeats, it represents another visit to the same provider. Both John and Ben have the same number of visits (7 visits), but their patterns of ambulatory care are different. John has one provider with a majority of visits, whereas Ben's visits are more diffusely spread across many providers, with no single provider having a substantial proportion of visits. Ben's care is more fragmented, and he has the higher rBBI. Icons by maradaisy and StockSmartStart; images reproduced with permission from Shutterstock.com.[39-41]

Appendix 4. Derivation of the study sample

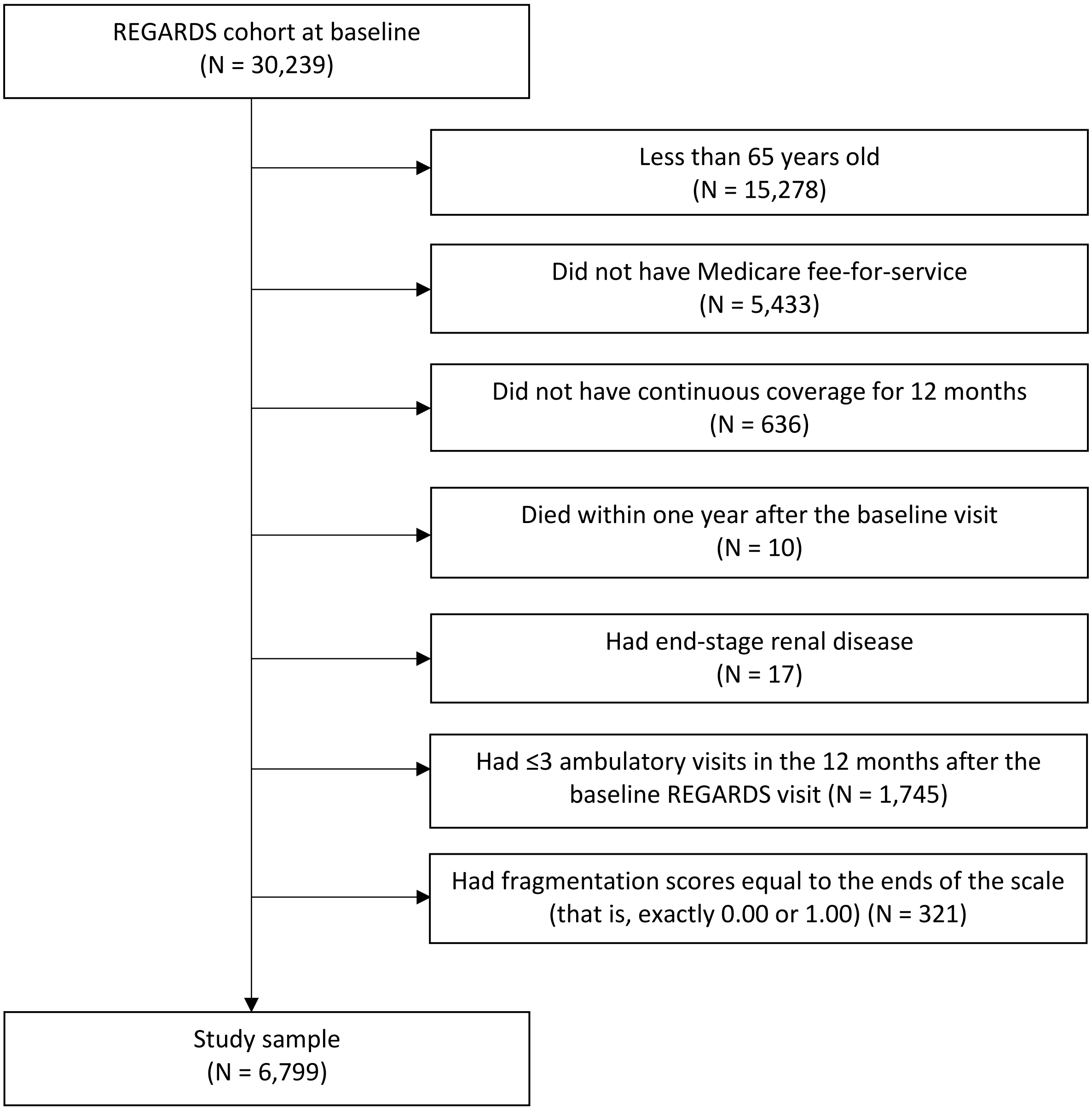

REGARDS = REasons for Geographic and Racial Differences in Stroke.

Appendix 5. Sensitivity analyses for the associations among race, income, education, and healthcare fragmentation

	Final model for primary analysis†	Sensitivity analysis 1‡	Sensitivity analysis 2§
Race<br>(black vs. white)	-0.027***<br>(-0.040,-0.015)	-0.033***<br>(-0.049, -0.017)	-0.049***<br>(-0.067,-0.031)
Annual household income<br>(<$35,000 vs. ≥$35,000)	-0.010<br>(-0.021,0.002)	-0.018*<br>(-0.032,-0.003)	-0.004<br>(-0.022,0.013)
Educational attainment<br>(high school or less vs. some college or more)	-0.002<br>(-0.013,0.009)	0.000<br>(-0.014, 0.014)	-0.030***<br>(-0.047,-0.013)

†Multivariable linear regression was used to determine associations among race, income, and educational attainment (as independent variables) and healthcare fragmentation (as a dependent variable, using the continuous reversed Bice-Boxerman Index). Negative coefficients indicate lower fragmentation scores than the reference group. Please see the Methods and Table 4 for more details.

‡Sensitivity analysis 1 is the same as the final model for the primary analysis, except that it re-included those with fragmentation scores equal to 0.00 or equal to 1.00 (N = 7,120).

§Sensitivity analysis 2 is the same as the final model for the primary analysis, except that the time period that the variables were drawn from differs. Sensitivity analysis 2 used co-variates from the second in-home visit and fragmentation scores from the 12 months following the second in-home visit (N = 2,166), instead of co-variates from the first in-home visit and fragmentation scores from the 12 months following the first in-home visit.

*$p < 0.05$. **$p \le 0.01$. ***$p \le 0.001$.

REFERENCES

1-38. See reference list in main manuscript.
39. StockStartSmart [artist]. Medical staff icons: Doctors and nurses medical staff avatars. 2018. (Accessibility confirmed February 15, 2021, at https://www.shutterstock.com/image-vector/medical-staff-icons-doctors-nurses-staffs-418733212.)
40. StockStartSmart [artist] Professional doctor avatars isolated on white background: Medicine professionals and medical staff people icons. 2018. (Accessibility confirmed February 15, 2021, at https://www.shutterstock.com/image-vector/professional-doctor-avatars-isolated-on-white-613984136.)
41. Maradaisy [artist]. Icons of elderly seniors. 2020. (Accessibility confirmed February 15, 2021, at https://www.shutterstock.com/image-vector/old-people-avatar-vector-person-flat-1663636021.)
